# Alternative ideas to increase the percentage of filled seats in nephrology fellowships

**DOI:** 10.12688/f1000research.3-3.v2

**Published:** 2014-01-13

**Authors:** Tejas Desai

**Affiliations:** 1 Division of Nephrology and Hypertension, East Carolina University, Greenville, NC, USA

## Abstract

Interest in nephrology has been decreasing for the last decade. In this opinion piece, the author provides four unconventional, outside-the-box strategies to increase the percentage of filled nephrology training positions.

## Introduction

Interest in nephrology fellowship positions has been decreasing since 2002. Data from the National Residency Match Program (NRMP) show an increasing number of nephrology fellowship positions but a decreasing number of applicants, primarily from those who have graduated from US medical schools ^1– 3^. On December 4, 2013, 24% of all nephrology positions in the United States were unfilled [NRMP data, unpublished]. This percentage represents the largest failure of nephrology recruitment and a steady worsening since 2002 ^1, 3^. In the early years, this decline in interest by US medical graduates (USMGs) was mitigated by an increase in the number of applications by foreign medical graduates (FMGs). In recent years, fewer FMGs have applied to nephrology fellowships, creating an additional strain amongst nephrology fellowship programs ^4^. Many reasons are offered to explain the declining interest in nephrology, each with their own merits and shortcomings. Common explanations include: 1) receiving lower salaries than other specialties ^2, 5^, 2) experiencing poor job prospects upon completion of nephrology training ^2, 5^, 3) working long hours ^2, 3, 5^, 4) caring for complicated, high acuity, and/or sick patients ^2, 3^, and/or 5) not witnessing positive breakthroughs in clinical research ^3^. Leaders in the field of nephrology have addressed these challenges by offering complimentary membership to leading nephrology organizations, deeply discounted journal subscriptions, and fully funded hands-on research opportunities. Though praiseworthy, these initiatives have been a universal failure. Because conventional methods have not resulted in an increase in applications, nephrology educators are now forced to think “outside-the-box” to increase interest in this field. In this paper, I offer four unconventional solutions to consider that may increase the percentage of filled nephrology fellowship positions.

## Idea 1: Offer combined nephrology-critical care fellowships positions

Anecdotal evidence suggests that applicants are increasingly attracted to critical care medicine ^6^. In an informal and unscientific survey of internal medicine residents and medical students, critical care medicine offers the following attractive features: 1) salaries that are well regarded by students and residents, 2) clearly defined work hours, and 3) excellent job prospects upon fellowship completion. At our institution, we have begun offering a one-year critical care medicine fellowship position to qualified candidates. Whether by chance or design, many applicants are requesting an integrated nephrology-critical care fellowship position. It is conceivable that combining nephrology with critical care training into a single 3-year consolidated fellowship may attract individuals who are “on the fence” regarding nephrology fellowship alone. The anticipated candidate for such a hybrid fellowship would be one who has a true scientific interest in nephrology and whose concerns about employment prospects, financial compensation, and/or the work hours can be alleviated by the critical care component of the training.

## Idea 2: Training programs should exit The Match

In 2009 nephrology fellowship programs entered the US National Residency Match Program (NRMP; The Match) ^7, 8^. The Match, which began in 1952, was intended to centralize the application process for graduate medical positions ^9^. The matching algorithm provides applicants with enough time to make a duress-free decision about the fellowship position they want ^8, 9^. One can debate whether applicants still need this protection, but it is increasingly evident that for unfilled nephrology training programs, The Match is harmful. Known as “the scramble”, unfilled nephrology programs enter into an unregulated, pressure-filled and disorganized ‘free-for-all’ to find individuals for their positions. Increasingly, nephrology programs are entering the scramble because the number of positions being offered (supply) is greater than the number of applicants (demand) ^4^. This ‘supply-demand’ imbalance places a select few programs in an undesirable, stress-filled environment in which they must fill training positions in the shortest amount of time. Put colloquially, the scramble allows any applicant of any quality, at the eleventh hour and from out of left field, to secure a nephrology fellowship position. The Match, whose intentions are to protect the applicants, actually selectively hurts nephrology fellowship programs. For those programs that cannot fill their training positions through The Match, the resulting scramble period opens their gates for un- or under-qualified applicants. Exiting The Match would return all nephrology fellowships to a pre-2010 state, in which every nephrology program would be on a level playing field. Each program would have greater control over recruitment. Indeed programs that exit The Match can develop innovative recruitment strategies; ones that are currently suppressed by The Match rules and cannot be executed in the scramble because of time constraints. Innovative recruitment would allow all nephrology programs to search for the *best* candidates instead of a few programs competing for the *available* candidates.

## Idea 3: Extend clinical nephrology fellowship training to three years

**Figure 1.**
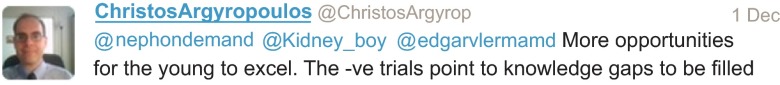
Nephrologist view of research. Dr. Argyropoulos is a nephrologist and pharmaceutical consultant in applied clinical research. He practices in Athens, Greece and can be reached on Twitter @ChristosArgyrop.

Extending clinical nephrology fellowship training to three years (from its current duration of two) is counterintuitive. Some programs offer 3-year research-track positions (less than 20% of all positions), but these are few and geared primarily to individuals who want a career in research ^7^. One would expect applicants to view a 2-year clinical training program more favorably than a 3-year program. The current 2-year duration has not been attractive enough to increase the number of applications to nephrology fellowship programs. Fellows in 2-year clinical training programs use their first year to develop their clinical skills and pass their American Board of Internal Medicine (ABIM) exam. In their second and final year, fellows focus on ABIM-specialty board preparations and searching for a job. As a result, 2-year clinical training programs offer limited time to experience the rewards of research and scholarly work. Many nephrology educators have considered the lack of research exposure and scholarly activities for fellows as having negative impacts on recruitment ^3^. Two-year clinical training programs may turn residents away who want to participate in a modest amount of research, but do not want a career heavily focused on it. The value of a 3-year training program is in its middle (second) year, where fellows can enhance their training by learning about and partaking in scholarly activities ( Figure 1). No longer having the pressures of taking and preparing for board exams, learning clinical nephrology and finding a job, fellows can enjoy research in a stress-reduced time period. Residents considering nephrology may now be attracted to training programs that will expose them to a greater degree of scholarly work in a less stressful environment.

## Idea 4: Decrease the number of positions offered

The most obvious and immediate solution is to limit the number of fellowship positions available. Smaller fellowship programs tremble at this idea. They believe that their small programs would be the most likely to shut down first, consistent with the commonly accepted but unproven rationale that the value of a fellowship program is directly proportional to its size. An alternative strategy exists that does not place the entire burden on small programs. Decrease the number of fellowship positions based on the geographic prevalence of chronic kidney disease (CKD) and/or end-stage renal disease (ESRD). Since the number of fellowship positions offered is loosely based on the anticipated need of kidney doctors, which in turn is based on the incidence and prevalence of CKD and ESRD in a geographic location, than any reduction in fellowship numbers should be based on these factors. Such a systematic method would ensure that programs would be cut based on the populations they serve and not based on their size, prestige, or other fellowship-specific factors. This method would be an equitable distribution of the pain associated with fellowship reduction and would not be prejudicial.

## Conclusion

In this exploration of potential policies, I have described four possible methods by which nephrology programs can decrease the percentage of unfilled fellowship positions. Although the effectiveness of these strategies has not been scientifically studied, each idea can be tested through a Plan-Do-Study-Act cycle (PDSA) ^10^. In a PDSA cycle, ideas can be tested on a small scale and temporary basis to determine outcomes. Those that result in unintended negative outcomes would not systemically affect all nephrology training programs when tested through a PDSA cycle. Because recruitment occurs yearly and the implementation of these ideas would require a major investment of resources, the PDSA cycle offers the best method by which these strategies can be tested.

Finally, these ideas do not address the disinterest that medical students and residents have for nephrology. Increasing interest would be the best and most long-lasting way in which to increase the number of applications to nephrology training programs. For years, national organizations and individual nephrology divisions have tried to cultivate a passion for nephrology in residents and medical students ^5^. As of 2013, however, those efforts have failed to increase the number of applications to our training programs. Perhaps more time is needed before one can determine if these endeavors have worked. In the interim, common sense ideas may need to be implemented to stave off the “tide” of disinterest in nephrology.
