# Supplementary material for: Alternative ideas to increase the percentage of filled seats in nephrology fellowships
Source: F1000Res. 2014 Jan 13;3:3. Originally published 2014 Jan 6. [Version 2] doi: 10.12688/f1000research.3-3.v2 (PMC3907163; doi:10.12688/f1000research.3-3.v2)
Supplement: Peer review history

# Peer review: Referee response for version 2

**Published:** 2014-01-27

**Authors:** Paul Phelan

**Affiliations:** 1 Department of Nephrology, Beaumont Hospital, Dublin, Ireland

Dr. Desai has written an article regarding novel ways to increase the number of unfilled Nephrology fellowship positions. This is a well written piece by an author who has a track record of interest and research in this area. His paper is timely, coming at a point where interest in the specialty is at an all-time low, and new ideas are sorely needed.

Specifically regarding his ideas:

1. Combined critical care fellowships: this seems a good idea on the face of it. However, do we have any evidence that trainees want to pursue a combined fellowship like this?
   
2. Programs exiting the match: While this will certainly help some programs, it is not realistic that competitive programs would ever consider this.
   
3. I personally think this is a good idea, as having been trained abroad where fellowship is longer I can see advantages to increased experience before exiting training. However, trainees on the most part would likely not be keen on this, and it may further decrease interest.
   
4. Decrease the number of positions. We certainly want quality applicants and it is not in anybody's interest to have substandard candidates getting positions just because there is a lack of demand. However this does not address the fundamental problem of why trainees are not choosing Nephrology.

Overall, this is a well written and thought-out article coming at a time of critical need for innovation to publicise Nephrology to trainees. While not everybody (including this reviewer) will agree with all the ideas in the paper, we need outside-the box thinking and innovative ideas so the editorial is welcome.

I have read this submission. I believe that I have an appropriate level of expertise to confirm that it is of an acceptable scientific standard.

# Peer review: Referee response for version 2

**Published:** 2014-01-13

**Authors:** Manish Ponda

**Affiliations:** 1 Laboratory of Biochemical Genetics and Metabolism, The Rockefeller University, New York, NY, USA

The article is well-written and I agree with many of Dr. Desai's points. The topic is highly relevant to the field of nephrology.

**A few comments/suggestions:**

For idea #1, it should be recognized that the critical care field will need to accept renal/critical care trainees into practice. If these combined-program trainees fail to find jobs, the combined program will eventual fail to attract trainees.

#2 - Exiting the Match could be voluntary; also programs should be able to have some spots off-Match .

#3 - Many programs already have NIH-sponsored T32 grants that allow for the support of a 3rd year of research. It is also common for programs to provide institutional support (non-NIH) for a 3rd year for interested candidates. Unfortunately, there is very little interest from prospective renal fellows even with these options available. Indeed, historically 3-year programs are now offering 2-year spots because of a lack of interest. Is there any data from NRMP to compare the success of 3-year vs. 2-year programs in terms of filling spots?  

The supply/demand imbalance may be inherent to nephrology itself, which I think Dr. Desai correctly identifies as a core issue in ideas #2/#4 and the Introduction.

I have read this submission. I believe that I have an appropriate level of expertise to confirm that it is of an acceptable scientific standard.
